# Cytotoxic and Bactericidal Effects of Inhalable Ciprofloxacin-Loaded Poly(2-ethyl-2-oxazoline) Nanoparticles with Traces of Zinc Oxide

**DOI:** 10.3390/ijms24054532

**Published:** 2023-02-25

**Authors:** Mohammad Zaidur Rahman Sabuj, Flavia Huygens, Kirsten M. Spann, Abdullah A. Tarique, Tim R. Dargaville, Geoffrey Will, Md Abdul Wahab, Nazrul Islam

**Affiliations:** 1 Pharmacy Discipline, School of Clinical Sciences, Faculty of Health, Queensland University of Technology, Brisbane, QLD 4000, Australia; 2 School of Biomedical Sciences, Faculty of Health, Queensland University of Technology, Brisbane, QLD 4000, Australia; 3 Centre for Immunology and Infection Control (CIIC), Faculty of Health, Queensland University of Technology, Brisbane, QLD 4000, Australia; 4 Child Health Research Centre (CHRC), The University of Queensland, Brisbane, QLD 4101, Australia; 5 School of Chemistry and Physics, Faculty of Science, Queensland University of Technology, Brisbane, QLD 4000, Australia; 6 School of Mechanical, Medical and Process Engineering, Faculty of Science, Queensland University of Technology, Brisbane, QLD 4000, Australia

**Keywords:** antimicrobial agents, antibiotic resistance, bactericidal effects, cytotoxicity, digestibility, inhalable formulation, nanoparticles, poly(2-ethyl-2-oxazoline), zinc oxide

## Abstract

The bactericidal effects of inhalable ciprofloxacin (CIP) loaded-poly(2-ethyl-2-oxazoline) (PEtOx) nanoparticles (NPs) with traces of zinc oxide (ZnO) were investigated against clinical strains of the respiratory pathogens *Staphylococcus aureus* and *Pseudomonas aeruginosa.* CIP-loaded PEtOx NPs retained their bactericidal activity within the formulations compared to free CIP drugs against these two pathogens, and bactericidal effects were enhanced with the inclusion of ZnO. PEtOx polymer and ZnO NPs did not show bactericidal activity alone or in combination against these pathogens. The formulations were tested to determine the cytotoxic and proinflammatory effects on airway epithelial cells derived from healthy donors (NHBE), donors with chronic obstructive pulmonary disease (COPD, DHBE), and a cell line derived from adults with cystic fibrosis (CFBE41o-) and macrophages from healthy adult controls (HCs), and those with either COPD or CF. NHBE cells demonstrated maximum cell viability (66%) against CIP-loaded PEtOx NPs with the half maximal inhibitory concentration (IC_50_) value of 50.7 mg/mL. CIP-loaded PEtOx NPs were more toxic to epithelial cells from donors with respiratory diseases than NHBEs, with respective IC_50_ values of 0.103 mg/mL for DHBEs and 0.514 mg/mL for CFBE41o- cells. However, high concentrations of CIP-loaded PEtOx NPs were toxic to macrophages, with respective IC_50_ values of 0.002 mg/mL for HC macrophages and 0.021 mg/mL for CF-like macrophages. PEtOx NPs, ZnO NPs, and ZnO-PEtOx NPs with no drug were not cytotoxic to any cells investigated. The in vitro digestibility of PEtOx and its NPs was investigated in simulated lung fluid (SLF) (pH 7.4). The analysed samples were characterized using Fourier transform infrared spectroscopy (ATR-FTIR), scanning electron microscopy (SEM), and UV–Vis spectroscopy. Digestion of PEtOx NPs commenced one week following incubation and was completely digested after four weeks; however, the original PEtOx was not digested after six weeks of incubation. The outcome of this study revealed that PEtOx polymer could be considered an efficient drug delivery carrier in respiratory linings, and CIP-loaded PEtOx NPs with traces of ZnO could be a promising addition to inhalable treatments against resistant bacteria with reduced toxicity.

## 1. Introduction

Lower respiratory tract infections (LRTIs) are a leading cause of morbidity and mortality globally. LRTIs associated with cystic fibrosis (CF) and chronic obstructive pulmonary disease (COPD) are commonly caused by bacterial pathogens, with the most frequently identified pathogens being *Pseudomonas aeruginosa* and *Staphylococcus aureus* [1]. Resistance to most antibiotics is growing for these pathogens in response to the current treatment being reliant on frequent administration of high doses of oral and intravenous (IV) antibiotics [2]. A more efficacious delivery system enabling the administration of lower-dose antibiotics would benefit patients suffering from LRTIs.

Pulmonary drug delivery technology enables the direct delivery of drugs to the site of respiratory infection. It produces superior bactericidal effects compared to the more commonly used oral or IV routes of the drug delivery [3]. Inhaled delivery of antibiotics guarantees a rapid onset of action, represents targeted drug delivery, and has practical therapeutic benefits at a relatively low dose of the drug with reduced costs [4].

Ciprofloxacin (CIP) is a second-generation synthetic fluoroquinolone antibiotic with broad-spectrum antibacterial activity against Gram-positive and Gram-negative bacterial pathogens [5]. Inhaled CIP provides advantages over high oral and IV administration doses by reducing systemic and dose-related side effects [6]. A phase I randomized, placebo-controlled clinical trial showed rapid absorption and clearance of drug-only inhaled formulations from the lungs [7]. In contrast, a drug encapsulated within a carrier, especially in polymers, prolongs drug release and enhances the therapeutic effects [8]. Carrier-based inhaled CIP has been investigated to identify its antibacterial efficacy against Gram-positive and Gram-negative bacteria and was found to be more effective than CIP-only formulations [8,9]. However, it is essential to ensure the safety profile of inhaled formulations in the respiratory system before they are given to patients with LRTIs. Inhaled CIP did not adversely affect healthy human volunteers in a clinical study [7]. Nevertheless, patients suffering from lung diseases and respiratory allergies were not included in this study and might not show the same effects. CIP has been reported to induce cytotoxic effects in several in vitro and in vivo experimentations on various cell types [10,11,12]. Therefore, the safety profile of the inhaled CIP must be confirmed before testing it in animal models and clinical settings. One of the most effective models for this is the primary airway epithelial cells [13].

Zinc oxide (ZnO) nanomaterial is a popular combination with antibiotics to increase the bactericidal effects. In combination with ZnO nanoparticles (NPs), conventional antibiotics demonstrated increased bactericidal effects with solid stability and improved cellular uptake by multidrug-resistant bacterial strains [14,15]. ZnO NPs, in combination with CIP, have shown enhanced bactericidal effects against *Escherichia coli*, *S. aureus*, and *Klebsiella* spp. at a very low dose in comparison to drug-only applications [16,17]. Moreover, ZnO nanocomposite in combination with other metal oxides demonstrated higher bactericidal activity against *K.* pneumonia, *S. aureus*, *Proteus vulgaris*, *E. coli,* and *P. aeruginosa* compared to standard CIP [18,19]. Furthermore, biosynthesized ZnO NPs demonstrated bactericidal effects against both Gram-negative and Gram-positive bacterial strains with biocompatibility on human erythrocytes [20]. ZnO NPs alone demonstrated considerable bactericidal effects against Gram-positive and Gram-negative bacteria [21]. Furthermore, ZnO NPs, combined with polymeric composites, demonstrated increased bactericidal effects, while smaller nanocomposites were found effective for cellular absorption [22]. ZnO NPs and their derivatives are also reported to show minimal toxic effects on mammalian cell lines [23]. Therefore, the inclusion of ZnO in combination with conventional antibiotics is a promising addition to the pulmonary drug delivery system.

The digestibility of a formulated polymer as a drug carrier is very important in order to determine the longevity of performance within biological systems. Due to this feature, the range of polymers that are effective as carriers in lung delivery is limited to only a few natural and synthetic polymers, including chitosan [24,25], alginate [26,27], poly(lactic-co-glycolic acid) (PLGA) [28,29], poly(lactic acid) (PLA) [30], poly(vinyl alcohol) [31,32], and polyethyleneimine [33]. Although these polymers are considered biodegradable because of their biodegradable by-products, the NPs of these polymers are yet to be determined as completely biodegradable or biocompatible [34]. Recently, our group investigated the enzymatic digestibility of glutaraldehyde cross-linked chitosan NPs in an in vitro method using lysozyme enzyme and found that cross-linked chitosan NPs were not digested in lysozyme, therefore compromising their applicability in pulmonary delivery [35]. Other studies found that cross-linked chitosan NPs were partially digestible in lysozyme enzymes [36,37,38,39]. However, the digestion mechanism of chitosan or its NPs is yet to be determined. Therefore, selecting digestible polymer drug carriers for pulmonary delivery is still a prominent field to study.

The application of minimal-toxic PEtOx polymer in biomedical fields is promising because of its biocompatible and highly soluble characteristics [40,41]. In this study, we have introduced PEtOx polymer for its potential applicability in pulmonary delivery. We developed and characterized inhaled CIP-loaded PEtOx NPs dry powder inhaler (DPI) formulation in terms of particle sizing, morphology analysis, crystallinity, chemical integrity, and thermal stability [42]. The formulations were also studied to determine the drug-loading and drug-release characteristics of the PEtOx polymer. We have recently investigated the stability of the CIP-loaded PEtOx NPs DPI formulation in highly stressed conditions [43]. In this present study, the bactericidal effects of the formulated CIP-loaded PEtOx NPs were determined compared to free CIP against *S. aureus* and *P. aeruginosa*. This study also investigated the bactericidal activity of CIP-ZnO-loaded PEtOx NPs against the same bacterial strains. Cytotoxicity of PEtOx NPs, CIP-loaded PEtOx NPs, and CIP-ZnO-loaded PEtOx NPs was studied on airway epithelial cells (AECs) as representative of structural cells and macrophages as representative of immune cells. Cytotoxicity was tested in AECs from healthy adult donors (NHBE), those with COPD (DHBEs) or CF (CFBE41o-) and macrophages from healthy controls (HCs), and CF-like macrophages. Inflammatory responses following CIP administration were also studied in HC and CF-like macrophages. Finally, the in vitro digestibility of PEtOx polymer and its NPs in simulated lung fluid (SLF) (pH 7.4) was tested to determine their applicability in lung delivery.

## 2. Results and Discussion

### 2.1. Synthesis of Blank, CIP and ZnO-Loaded PEtOx NPs

A straightforward co-assembly reaction was utilized to synthesize the blank, CIP-loaded PEtOx NPs, as described in our previously published article [42]. Size uniformity was ensured by the predetermined concentration of the tannic acid (TA) (0.03%) and PEtOx (1%) solutions in deionized water and the stirring speed (1000 rpm) while mixing the components. The average particle sizes of the blank NPs, ZnO, and the CIP-ZnO-PEtOx NPs were 151.6 ± 1.7 nm, 157.5 ± 4.5 nm, and 176.0 ± 3.5 nm, respectively. The sizes of the NPs increased with ZnO and CIP loading into the nanocomposites, and the CIP-ZnO-PEtOx NPs showed the largest particle sizes.

### 2.2. X-ray Photoelectron Spectroscopy (XPS) Analysis of ZnO-Loaded PEtOx NPs

X-ray Photoelectron Spectroscopy (XPS) analysis was carried out to confirm the presence of Zn within the nanocomposites. Two weak peaks at 1041 eV for Zn 2P_1/2_ and 1020.7 eV for Zn 2P_1/2_ (Figure S1) were obtained (Figure S1) and supported by others [44,45]. This outcome demonstrated approximately 2% Zn loading within the developed NPs. This amount of Zn could be considered as the trace amount of Zn within the CIP-ZnO-loaded PEtOx NPs.

### 2.3. Bactericidal Effects of Blank PEtOx NPs and CIP-Loaded PEtOx NPs

**Figure 1:**
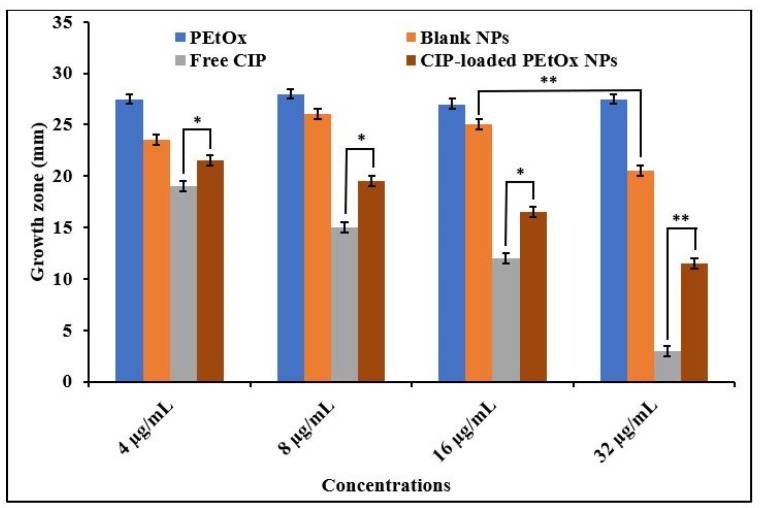
Bactericidal effects of PEtOx, blank NPs, free CIP, and CIP-loaded PEtOx NPs against *S. aureus*. *p* > 0.05 (*), *p* < 0.05 (**).

**Figure 2:**
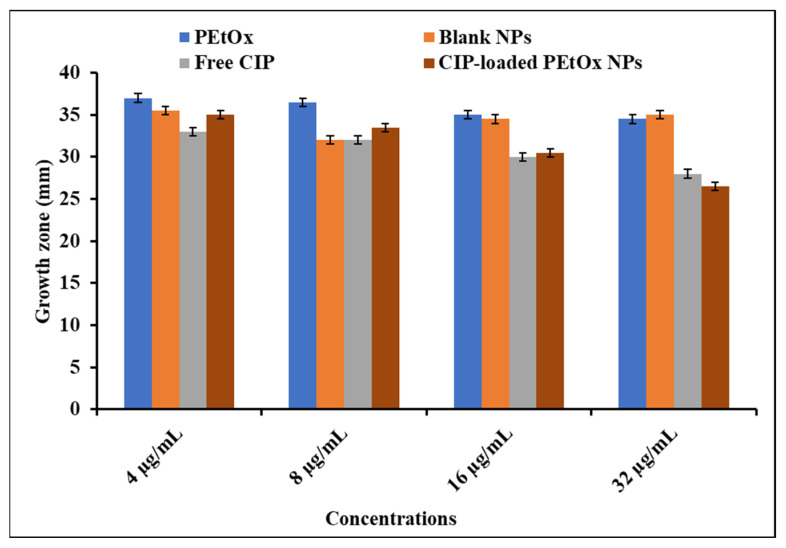
Bactericidal effects of PEtOx, blank NPs, free CIP, and CIP-loaded PEtOx NPs against *P. aeruginosa*.

The findings of growth zone diameter measurement for *S. aureus* and *P. aeruginosa* against the applied compounds are presented in Figure 1 and Figure 2, respectively. CIP alone and CIP-loaded PEtOx NPs demonstrated a slightly high bactericidal effect against *S. aureus* than *P. aeruginosa* (*p =* 0.024). The increased bactericidal activity of CIP against a Gram-positive bacterium was likely due to the absence of the lipid layer in the cell membrane. In contrast, Gram-negative bacteria have an outer lipid membrane. The lipid layer on the outer membrane prevents the penetration of antibiotics inside the cell wall of Gram-negative bacteria and reduces the antibacterial effects of the applied antibiotics [46]. The concentrations of free CIP compared to CIP released from PEtOx NPs (4–16 µg/mL) did not result in significant differences (*p >* 0.05) in the growth of the bacterial strains. This indicates that CIP maintained its chemical integrity after drug loading and drug release from formulated NPs. Similar findings were also reported by Hua et al. [47] using CIP-loaded PLGA micro/NPs against *P. aeruginosa* to determine the chemical integrity of CIP after encapsulation and drug release. However, we did observe a difference in the growth zone of *S. aureus* treated with 32 µg/mL of free CIP compared to that of CIP-released from PEtOx NPs (Figure 1; *p* < 0.05), indicating that CIP might not be released entirely from the PEtOx polymer after seven days as demonstrated in our previously published article [42]. Similar bactericidal effects were observed against *P. aeruginosa* when CIP-loaded alginate/chitosan NPs were investigated to determine the relationship between the antimicrobial effects of the CIP-loaded NPs, free CIP, and NPs without the drug [21]. Therefore, these data demonstrated that CIP maintained its potency within the formulated NPs after the drug encapsulation and drug release from the polymeric matrix.

PEtOx polymer did not show any bactericidal effects against bacterial strains compared to the bacterial growth on the Mueller–Hilton agar (MHA) medium without any treatment. Thus, PEtOx polymer itself does not affect bacterial growth, which was also reported by Abilova et al., 2020 [48]. Blank NPs also demonstrated no bactericidal effects against any bacterial strain except 32 µg/mL blank NPs against *S. aureus*. Blank NPs showed significant (*p* = 0.021) bactericidal effects at this concentration compared to PEtOx polymer. This might happen due to the increased concentration of TA within the blank NPs, as TA itself has been reported as having bactericidal effects against Gram-positive and Gram-negative bacteria [49]. However, in this study, blank NPs did not show bactericidal effects against the clinical strain of *P. aeruginosa*, which indicates that TA has a lower level of bactericidal effects against the clinical strain of Gram-negative bacteria.

Multiple studies have shown that the bactericidal effects of CIP are improved when encapsulated within polymeric NPs, as this allows penetration of the bacterial cell wall [50,51]. However, we did not observe enhanced bactericidal effects of CIP when encapsulated within polymeric NPs. Nevertheless, CIP retained its potency against the bacterial strains tested here.

In this study, the bactericidal effects of raw PEtOx, blank NPs, and CIP-loaded NPs were also studied to evaluate the antimicrobial activity of PEtOx polymer and determine the integrity of CIP within the formulated NPs. Free CIP was also reviewed to compare with the NPs and determine whether nanofabrication impacts the potency of CIP. The findings revealed that CIP retained its antibacterial potency within the formulations, and the polymer did not increase the drug’s potency. However, blank PEtOx NPs may have some bactericidal effects against Gram-positive bacteria (Figure 1).

### 2.4. Combined Effect of CIP-ZnO-Loaded PEtOx NPs

**Figure 3:**
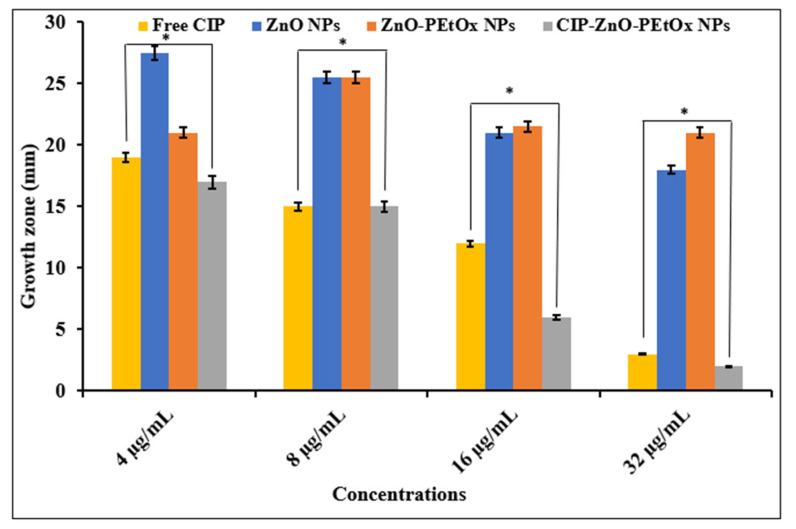
Bactericidal effects of free CIP, ZnO NPs, ZnO-PEtOx NPs, and combination of CIP-ZnO-loaded PEtOx NPs against *S. aureus*. *p* > 0.05 (*).

**Figure 4:**
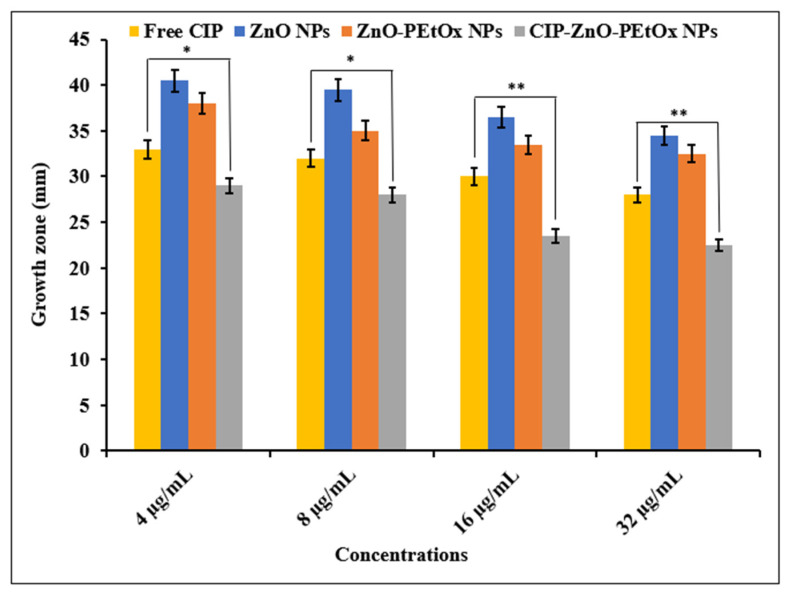
Bactericidal effects of free CIP, ZnO NPs, ZnO-PEtOx NPs, and combination of CIP-ZnO-loaded PEtOx NPs against *P. aeruginosa*. *p* > 0.05 (*), *p* < 0.05 (**).

The combined effects of ZnO and CIP-loaded PEtOx NPs were evaluated against both bacterial strains (Figure 3 and Figure 4). The enhanced bactericidal effects of CIP-ZnO-loaded PEtOx NPs were observed against all the bacterial strains compared to that of CIP-loaded PEtOx NPs. In addition, CIP-ZnO-loaded PEtOx NPs demonstrated significantly (*p* < 0.05) improved bactericidal effects compared to free CIP against *P. aeruginosa* and moderately improved bactericidal effects against *S. aureus* (*p* = 0.740). However, CIP-ZnO-loaded PEtOx NPs demonstrated maximum inhibitory activity against *S. aureus* at 32 µg/mL concentration. The enhanced bactericidal effects occurred due to the presence of ZnO in CIP-ZnO-loaded PEtOx NPs compared to free CIP and CIP-loaded PEtOx NPs. ZnO is usually reported to form hydroxy radicals by its photochemical activity in combination with antibiotics and the polymer matrix against the bacterial cell wall [22]. The bacterial cell membrane would have been significantly damaged due to the combined effects of the traces of Zn in the CIP-ZnO-loaded PEtOx nanocomposites. This may result in easier penetration of the bacterial cell wall by antibiotics and significantly reduced bacterial growth. Scientists have reported similar enhanced bactericidal effects of ZnO NPs in combination with CIP against Gram-positive bacteria [15,16,17]. However, the same concentration did not perform accordingly against *P. aeruginosa* compared to *S. aureus*, potentially due to the presence of the outer membrane layer and a much thinner peptidoglycan layer of the cell wall. Similar findings of lower bactericidal effects of ZnO-CIP NPs against *P. aeruginosa* were reported by the scientist compared to Gram-positive *S. aureus* and *Bacillus cereus* bacteria [21].

ZnO NPs and ZnO-PEtOx NPs showed no significant bactericidal (*p* > 0.05) effects against any bacterial strains compared to the CIP-loaded composites. In a previous study, ZnO did not show antibacterial activity against *S. aureus* and *E. coli* [16]. In another study, ZnO NPs demonstrated significant bactericidal effects against *S. aureus* and foodborne bacteria, including *Salmonella typhimurium* and *B. cereus* [21]. In this study, ZnO-PEtOx NPs were also found to show minimal bactericidal effects against the clinical bacterial strains; however, the addition of traces of Zn with CIP in the polymer nanoparticles showed excellent antibacterial effects (Figure 3 and Figure 4). Therefore, the CIP-ZnO-PEtOx nanocomposite is a promising bactericidal agent against the clinical strains of lung pathogens. The findings also revealed that the CIP-ZnO-PEtOx NPs were more active against the Gram-positive bacteria than the Gram-negative bacteria.

### 2.5. Cellular Viability by Lactate Dehydrogenase (LDH) Assay

**Figure 5:**
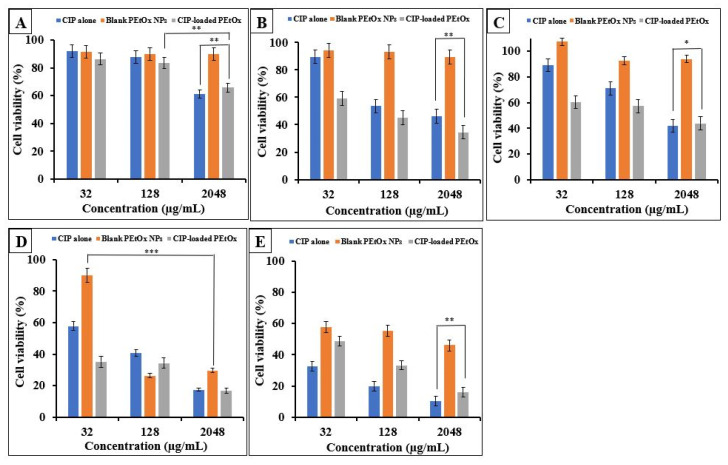
Effect of CIP alone, blank PEtOx NPs, and CIP-loaded PEtOx NPs on cell viability (%) of (**A**) NHBE cells, (**B**) DHBEs, (**C**) CFBE41o- cell line, (**D**) HCs macrophages, and (**E**) CF-like macrophages. *p* > 0.05 (*), *p* < 0.05 (**), *p* < 0.001 (***).

**Figure 6:**
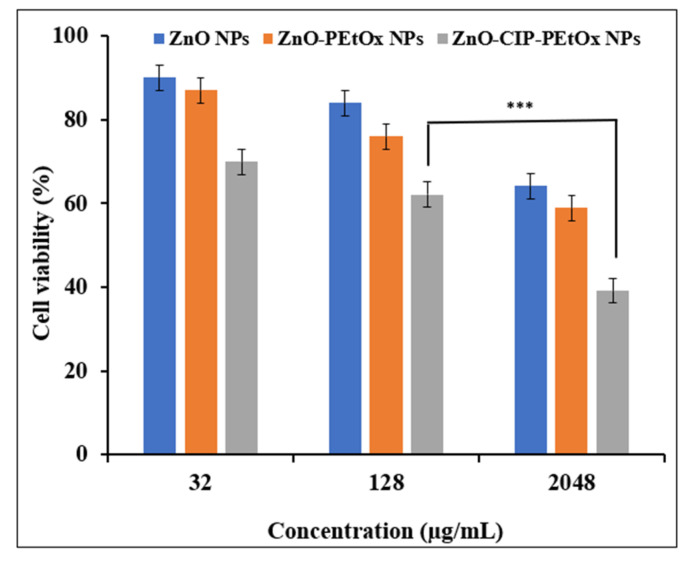
Effect of ZnO NPs, ZnO-PEtOx NPs, and CIP-ZnO-PEtOx NPs on the cell viability (%) of NHBE cells. *p* < 0.001 (***).

The cytotoxicity of PEtOx polymer and its various derivatives was determined on NHBE, DHBEs, CFBE41o- cell line, macrophages from HCs, and CF-like macrophages after 24 h exposure at different concentrations (Figure 5 and Figure 6). Lactate Dehydrogenase (LDH) release indicated that NHBE cells retained the highest viability against the CIP-loaded PEtOx NPs at 32 µg/mL (86% cell viability). However, increased concentrations of CIP-loaded PEtOx NPs (2048 µg/mL) decreased the cell viability of NHBEs (*p* > 0.05) (Figure 5A). The half maximal inhibitory concentration (IC_50_) of CIP-loaded PEtOx NPs on the NHBE cells was 50.7 mg/mL, which indicates very low cytotoxicity. CIP alone also demonstrated some cytotoxicity on NHBEs. However, CIP-loaded PEtOx NPs significantly increased (*p* < 0.05) cell viability at 2048 µg/mL concentration (Figure 5A). This indicated that the amount of PEtOx increased with the increasing concentration of CIP-loaded PEtOx NPs and demonstrated less toxicity, which has also been reported by others [12]. The findings also revealed that the PEtOx polymer is less toxic to the NHBE cells (90% cell viability), as reported using prostate epithelial cells and cancer cells [52]. CIP alone and CIP-loaded PEtOx NPs demonstrated more cytotoxic effects on DHBEs (Figure 5B) than NHBEs for all concentrations tested (IC_50_ 0.103 mg/mL for CIP-loaded PEtOx). However, blank PEtOx NPs did not show significant cytotoxicity on NHBE (Figure 5A), DHBEs (Figure 5B), and CFBE41o- (Figure 5C) cell lines.

Inhalable drugs are usually formulated with active ingredients and excipients less than a hundred micrograms in total weight [53]. Thus, the formulation of CIP-loaded PEtOx NPs at low concentrations could be considered safe inhalation, considering the low-level cytotoxic effects on COPD patients. The findings also revealed that blank PEtOx NPs are not cytotoxic on the DHBEs (89% cell viability) (Figure 5B). Thus, the cytotoxic effects of the CIP-loaded PEtOx NPs are derived from the CIP drug rather than the PEtOx polymer [10]. A low level of cytotoxicity of CIP-loaded PEtOx NPs was also noticed on the CFBE41o- cell line (IC_50_ 0.514 mg/mL). PEtOx polymer was also found to show no cytotoxic effects on the CFBE41o- cell line (90% cell viability) (Figure 5C). However, concentration-dependent cytotoxicity revealed a low dose of CIP-loaded PEtOx NPs might be safe for CFBE41o- cells.

Macrophages are the first line of the immune defence [54]. To investigate whether the formulations could induce inflammation, these formulations were tested in macrophages from HC as representative of homeostasis and CF-like macrophages as representative of a diseased condition. Macrophages from HCs and CF-like macrophages demonstrated a high level of cytotoxicity against the CIP-loaded PEtOx NPs (Figure 5). IC_50_ values of HCs and CF-like macrophages were 0.002 mg/mL and 0.021 mg/mL, respectively. IC_50_ values less than 0.01 mg/mL are considered highly cytotoxic in pharmaceutical applications [53]. Thus, a significant number of macrophages will be killed and might not be able to respond against inhaled CIP-loaded PEtOx NPs DPI formulation. However, it is prevalent for lung macrophages to provide an innate immune response against pathogens or foreign particles; therefore, the findings reveal that the formulations might escape the alveolar defence mechanism and reach into the deep lungs [55]. Moreover, the current formulations could be modified in various shapes to penetrate the mucus layer to overcome the clearance challenges by the alveolar macrophages [56].

In this study, ZnO was extensively used to understand the synergy of ZnO and CIP-loaded PEtOx NPs against respiratory pathogens. The findings demonstrated excellent bactericidal effects against clinical strains of Gram-positive and Gram-negative bacteria. Thus, it is also essential to determine the biological safety profile of the synergy on the airway epithelial cells. Therefore, ZnO NPs, as supplied by the manufacturer, ZnO-PEtOx NPs, and CIP-ZnO-PEtOx NPs, were studied on the NHBE cells, and cell viability was determined by LDH release. The findings from the LDH assay are presented in Figure 6. The results revealed that cell viability was reduced significantly (*p* < 0.05) with the increasing concentration of ZnO NPs. Other researchers also reported a similar dose-dependent cell viability reduction when ZnO was tested on lung epithelial cells [57,58]. However, the IC_50_ value demonstrated that ZnO NPs did not have notable cytotoxic effects on the NHBE cells (IC_50_ = 21.09 mg/mL). Similar negligible-toxic results of ZnO NPs were reported by Stankovic et al. [23] on human hepatoma (HepG2) cells. Moreover, ZnO-PEtOx NPs also demonstrated minimal-toxic effects on the NHBE cells (IC_50_ 7.52 mg/mL). Similar findings were also reported by Stankovic et al. [23] when they studied PLGA/nano ZnO NPs on the hepatoma cells.

CIP-ZnO-PEtOx NPs also demonstrated minimal-toxic effects on the NHBE cells (IC_50_ 0.52 mg/mL). However, CIP-ZnO-PEtOx NPs demonstrated significantly reduced (*p* < 0.05) cell viability compared to ZnO NPs and ZnO-PEtOx NPs. According to our findings on the cytotoxicity of CIP alone, the cytotoxicity of CIP-ZnO-PEtOx NPs occurred due to the addition of CIP within the formulations rather than the drug-free NPs. However, inhaled low-dose drugs in combination with ZnO NPs would be a promising addition to improving pulmonary delivery.

### 2.6. Proinflammatory Effects

**Figure 7:**
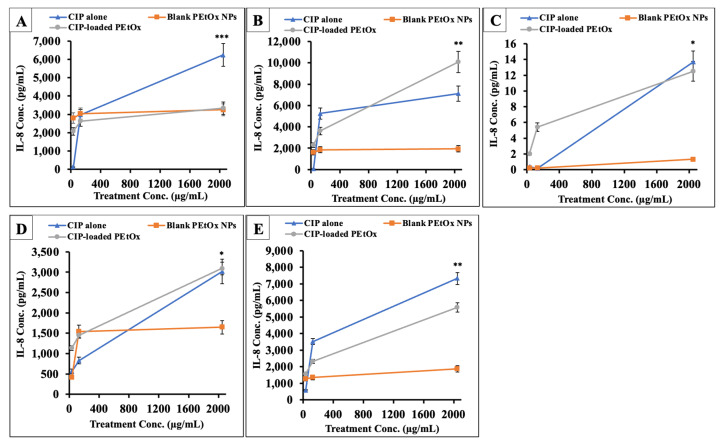
Proinflammatory effects of CIP alone, blank PEtOx NPs, and CIP-loaded PEtOx NPs on (**A**) NHBE cells, (**B**) DHBEs, (**C**) CFBE41o-cell line, (**D**) HCs macrophages, and (**E**) CF-like macrophages. The IL-8 secretion levels were normalized with the number of cells by dividing the % cell viability estimated by the LDH assay. Results expressed as the mean ± SD (*n* = 3) and *p* > 0.05 (*), *p* < 0.05 (**), *p* < 0.001 (***).

The compounds were also assessed for Interleukin-8 (IL-8) release to understand inflammatory effects on AECs and macrophages (Figure 7). IL-8 release by NHBE cells in response to CIP-loaded PEtOx NPs was between 2070 to 3342 pg/mL, which is higher than the basal limit of IL-8 releases (2290 pg/mL) of NHBE cells. However, the maximum IL-8 release was observed with a 2048 µg/mL concentration, slightly higher than the concentration of the inhalable dose. However, DHBE cells secreted 2298 to 10,074 pg/mL IL-8 against the formulation, which is 2- to 7-fold higher than the basal limit of IL-8 secretion (1453 pg/mL) by DHBE cells. Thus, the formulation might produce some proinflammatory effects on the DHBE cells. However, DHBE cells secreted no more than 1936 pg/mL IL-8 against PEtOx blank NPs regardless of dose, suggesting that CIP-induced IL-8 production and the polymeric complex alone did not [10]. Similar findings were also observed on CFBE41o- cell line. However, low concentrations of CIP-loaded PEtOx NPs initiated no more than six pg/mL IL-8 release from the CFBE41o- cell line. The previous literature reported that CIP itself did not inhibit the IL-8 secretion from the infections caused by *P. aeruginosa*, while azithromycin was found to reduce IL-8 release significantly [59]. In addition, CIP alone produces a very high level of IL-8 compared to CIP-loaded PEtOx NPs (Figure 7C). Therefore, the proinflammatory effect was initiated by the drug rather than the polymer or its composites. The proinflammatory cytokines initiated the increased IL-8 production against the inflammatory reactions due to the increased concentrations of CIP. The increased IL-8 also played an essential role in the activation of neutrophils for repairing damaged or inflamed tissues [60].

CF-like macrophages produced approximately 2-fold high IL-8 compared to the HCs macrophages (Figure 7D,E). However, this is not significantly modulated by CIP-loading into the PEtOx NPs. Therefore, as we discussed above, further studies are warranted to understand the safety profile of the formulated CIP-loaded PEtOx NPs and their applicability in pulmonary delivery.

### 2.7. Attenuated-Total Refection-Fourier Transform Infrared (ATR-FTIR) of the Digestion Studies

#### 2.7.1. PEtOx Polymer Digestion

**Figure 8:**
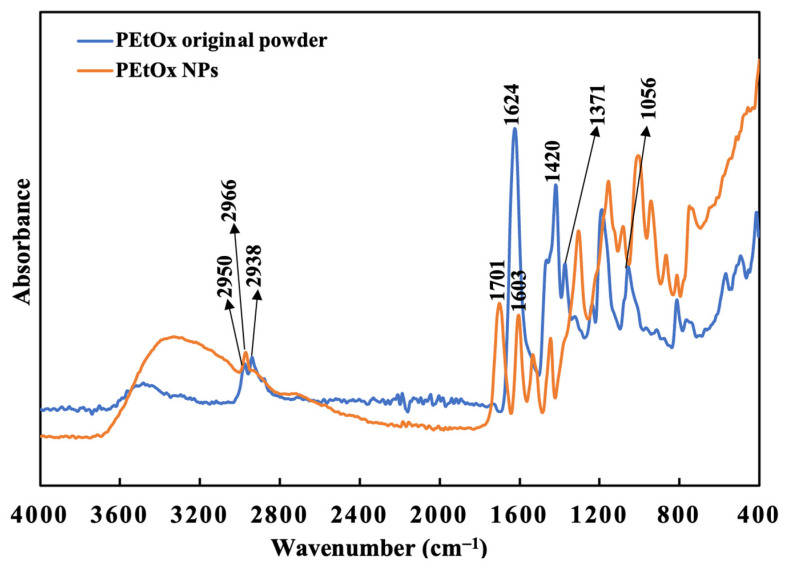
FTIR spectra of PEtOx powder and PEtOx NPs before incubating them in SLF.

**Figure 9:**
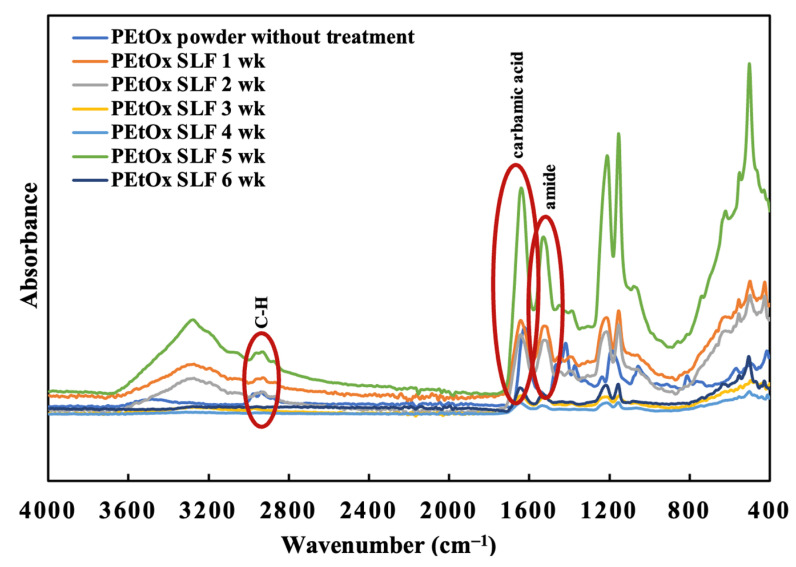
FTIR spectra of PEtOx powder before and after the incubation period of six weeks in SLF.

PEtOx original powder and NPs were analysed through Fourier Transform Infrared (FTIR) spectra to determine the digestibility of the polymer and its NPs in SLF (pH 7.4) at 37 °C. PEtOx original polymer was also analysed to compare its characteristic peaks with the samples after digestion. PEtOx original powder demonstrated two characteristic peaks for overlapping stretching vibration of C-H at 2938 cm^−1^ and 2950 cm^−1^ in the spectral analysis (Figure 8). Moreover, the typical characteristic peaks of the amide carbonyl band and carbamic acid vibration (C=O) were recorded at 1420 cm^−1^ and 1624 cm^−1^, respectively [42]. C-H bending vibration was recorded at 1371 cm^−1^, and the C-C stretching vibration was recorded at 1187 cm^−1^ and 1056 cm^−1^ [52]. A minor shift of the C-H stretching vibration was observed from 2938 cm^−1^ to 2917 cm^−1^, and the overlapping stretching band at 2950 cm^−1^ appeared weak after one week of incubation (Figure 9). However, no changes in other characteristic peaks occurred. The C-H stretching vibration was recorded at 2962 cm^−1^, and the overlapping stretching C-H vibration disappeared after three weeks of incubation (Figure 9). However, this does not indicate the digestion of the polymer, as the C-H stretching vibration was still present. The PEtOx powder did not produce new peaks at the end of 6 weeks, indicating that the polymer might not be digestible in lung fluids. Similar stable FTIR spectra were also reported by Putra et al. [61] when they used PEtOx as the coating of the additively manufactured biodegradable porous iron. Although the layer significantly enhanced the porous iron’s biodegradation rate, the polymer remained stable for 28 days.

#### 2.7.2. PEtOx NPs Digestion

The FTIR spectra of PEtOx NPs were recorded before incubating them in the SLF medium at pH 7.4. The findings are presented in Figure 8. The NPs were formed by the hydrogen bonding between TA and the PEtOx polymer. Thus, several characteristic peaks of hydrogen bonding appeared in the FTIR spectra of PEtOx NPs. The intensive peak of hydrogen bonding of -C=O was recorded at 1603 cm^−1^ and 1701 cm^−1^ [42].

**Figure 10:**
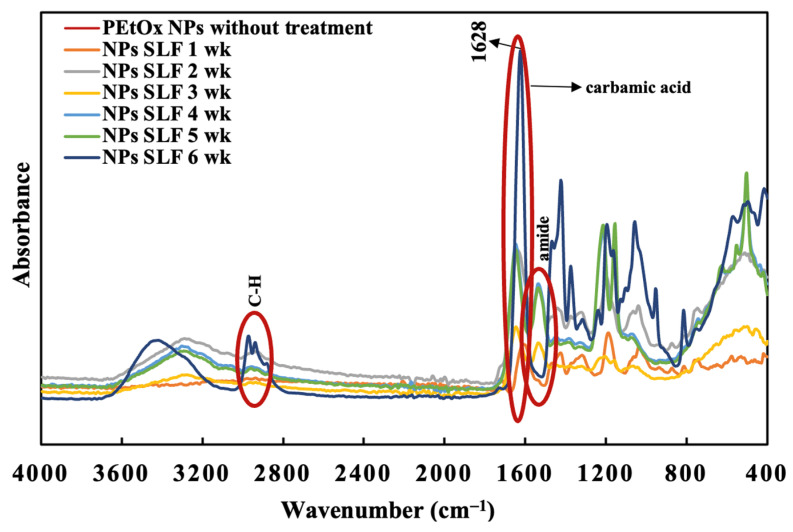
FTIR spectra of PEtOx NPs before and after the incubation period of six weeks in SLF.

PEtOx NPs also demonstrated a single C-H stretching vibration at 2966 cm^−1^ in place of two overlapping stretching vibrations of PEtOx powder. The hydrogen bonding at 1701 cm^−1^ disappeared, and a new peak appeared at 1717 cm^−1^ after one week of incubation, which is the characteristic peak of TA according to the previous findings (Figure 10) [62]. Thus, the FTIR spectra confirmed that the NPs break down in SLF after one week and return to their original form of TA. Moreover, the newly appeared peaks of the incubated samples at 1717 cm^−1^ disappeared after two weeks of incubation (Figure 10). Thus, TA is digestible in lung fluid and safe for pulmonary delivery. Additionally, the carbamic acid vibration (C=O) of PEtOx NPs shifted from 1603 cm^−1^ to 1632 cm^−1^, which is very close to the carbamic acid vibration (C=O) peaks of the PEtOx powder at 1624 cm^−1^ (Figure 8). This indicates that PEtOx NPs are returning to their original form PEtOx powder after two weeks of incubation. In addition, the reappearance of the amide carbonyl band at 1420 cm^−1^ confirms that the polymer retained its original form after the degradation of the PEtOx NPs. However, no more changes appeared in the FTIR spectra after the completion of the incubation period at the end of week six.

This digestibility study revealed that the PEtOx NPs are digestible in lung fluids and applicable in pulmonary delivery. This excellent feature encourages scientists to prepare biodegradable micelles using PEtOx for drug delivery [63]. However, PEtOx itself is not digestible in lung fluids; thus, it is essential to determine its biocompatibility before applying the polymer and its formulations in clinical studies. PEtOx is also reported to be acid-sensitive at pH 5.0 [63]. Thus, further studies are warranted to understand the complete digestibility of the polymer and its formulations in biological systems.

### 2.8. Morphology Studies by Scanning Electron Microscopy (SEM)

**Figure 11:**
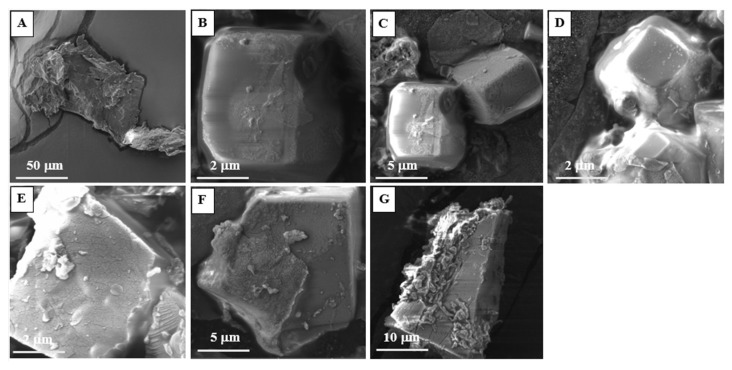
SEM photomicrographs of PEtOx powder before and after incubating in SLF at different time intervals (**A**) PEtOx original powder (**B**) PEtOx 1 week (**C**) PEtOx 2 weeks (**D**) PEtOx 3 weeks (**E**) PEtOx 4 weeks (**F**) PEtOx 5 weeks (**G**) PEtOx 6 weeks.

**Figure 12:**
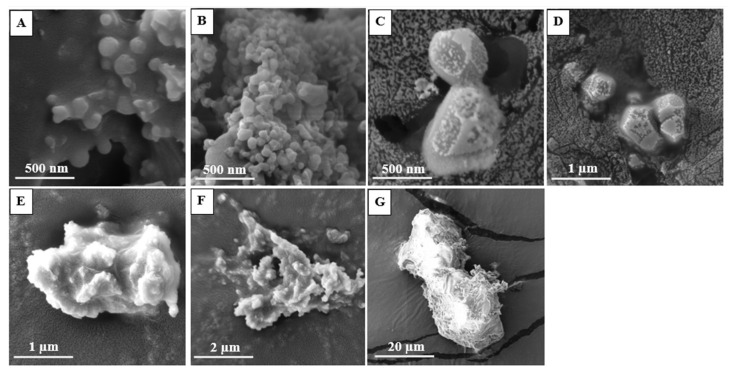
SEM photomicrographs of PEtOx NPs before and after incubating in SLF at different time intervals (**A**) PEtOx original NPs (**B**) PEtOx NPs 1 week (**C**) PEtOx NPs 2 weeks (**D**) PEtOx NPs 3 weeks (**E**) PEtOx NPs 4 weeks (**F**) PEtOx NPs 5 weeks (**G**) PEtOx NPs 6 weeks.

Scanning electron microscopy (SEM) images determined the morphology of the PEtOx powder and the NPs before and after the incubation period. PEtOx original powder did not show any noticeable morphological changes after the incubation period (Figure 11). However, the polymer powders appeared to be larger with smooth surfaces compared to the original PEtOx powder after one week. This confirms that the polymer is not digestible in lung fluids, which matches the findings of the FTIR spectra. PEtOx NPs appeared to stay similar after one week in morphology and particle size (Figure 12B). However, after two weeks of incubation, the NPs disappeared entirely, and some rough surface particle-like shapes appeared (Figure 12C), which might be the crystal structure of the PEtOx polymer [64]. After two weeks of incubation, the incorporation of NPs into the original polymer also matches the FTIR spectra. The polymer returned to its original shape after four weeks of the incubation period (Figure 12E). Finally, the NPs’ morphology returned to the original surface properties of the polymer powder after six weeks of the incubation period (Figure 12A,G). The digestion of NPs might also have occurred due to the Ostwald digestive ripening effect by replacing the existing hydrogen bond between the weak acid and the polymer and retaining its polymeric structure [65]. Therefore, the findings from the SEM images also confirm that the PEtOx NPs are digestible in SLF at pH 7.4. However, the PEtOx polymer itself is not digestible in the lung fluids and retains its stability for a long time, which also matches previous findings [61].

### 2.9. UV–Vis Spectroscopy

**Figure 13:**
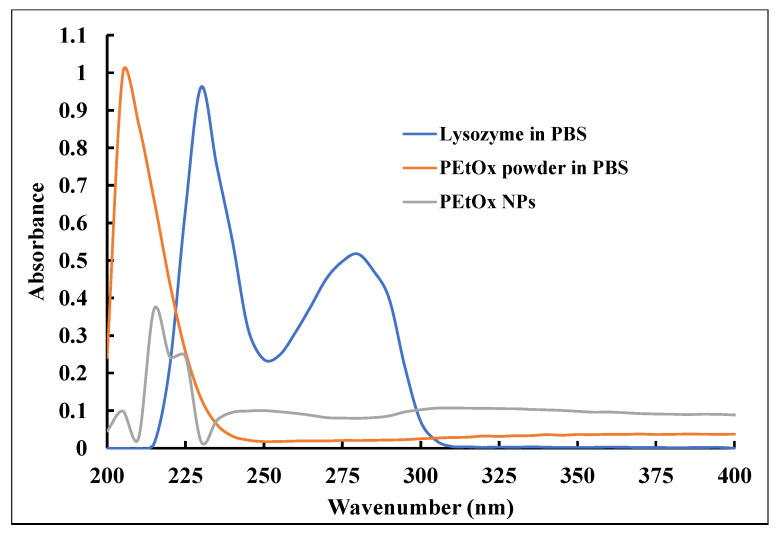
UV–Vis spectra of PEtOx solution (16 µg/mL) in PBS, PEtOx NPs in PBS (16 µg/mL), and lysozyme solution (0.2 mg/mL) in PBS (pH 7.4) PBS was used as the reference.

UV–Vis spectroscopy was used to determine the characteristic peaks of powder PEtOx polymer, PEtOx NPs, and the liquid samples analysed in the digestibility study. PEtOx powder dissolved in phosphate-buffered saline (PBS) solution demonstrated a single sharp peak at 205 nm as the absorbance value in the UV–Vis spectral analysis (Figure 13). Furthermore, PEtOx NPs demonstrated a sharp peak at 215 nm and a weak peak at 225 nm. However, another very weak peak was also visible at 205 nm, which resembles the characteristic peak of the PEtOx powder and indicates there might be some free polymer within the formulations. The original lysozyme dissolved in PBS solution produced two characteristic absorbance peaks at 230 nm and 278 nm, which did not match the peaks of the PEtOx polymer. This finding ensured that the lysozyme did not interfere with the characteristic peaks of the polymer (Figure 13). However, the UV–Vis spectra of lysozyme solution tend to approach the negative absorbance, which is usually considered the impurities of the molecules [66]. The negative absorbance phenomenon is also described as the light sensitivity of the samples and shifting from ground states to lower molecular orbitals or from ground states to excited states and then back to lower molecular orbitals. A similar phenomenon of negative absorbance in UV–Vis spectra from lysozyme solution was observed in one of our previous studies [35], which is also supported by others.

**Figure 14:**
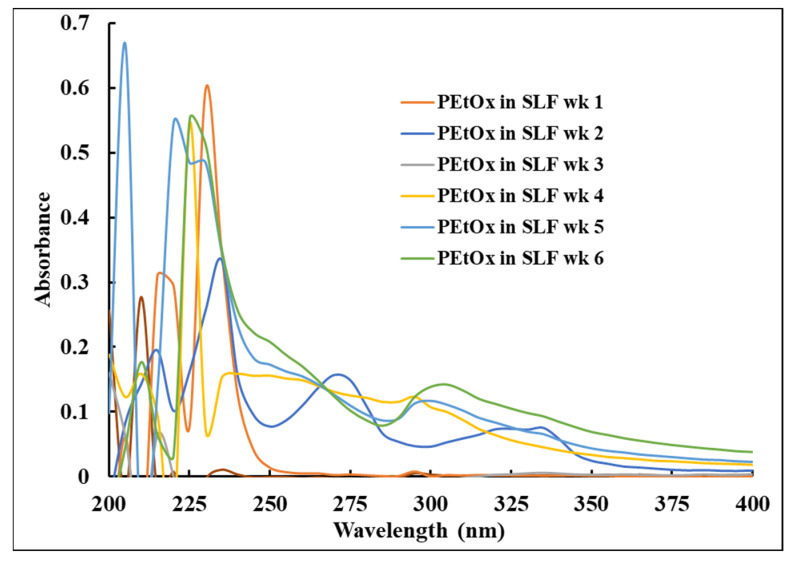
UV–Vis spectra of PEtOx powder at different time intervals incubated in SLF. SLF was used as the reference.

Figure 14 represents the UV–Vis of PEtOx powder incubated in SLF at various time intervals. After one week of incubation, the characteristic peaks of the PEtOx powder remained at the same position at 205 nm with low intensity. However, two more peaks appeared at 220 nm (weak) and 230 nm (sharp). The sharp peak resembles one of the characteristic lysozyme peaks, indicating an insignificant amount of PEtOx powder dissolved in SLF in the first week. Similar findings were also observed throughout the study, and the characteristic peak of the PEtOx powder keeps stable in the same position. Similar stable characteristics of PEtOx polymer at pH 7.4 was also reported previously [63]. This indicates that SLF is not capable of digesting the PEtOx polymer. The negative absorbance phenomenon of the spectral analysis might be originated from the lysozyme solution or from the impurities of the degraded NPs after the incubation period [67,68]. We used polymer nanoparticles, which were not completely degraded, and the samples were not filtered out after centrifugation, which might have left some fine particles that affected the light transmission and resulted in obtaining negative absorbance.

**Figure 15:**
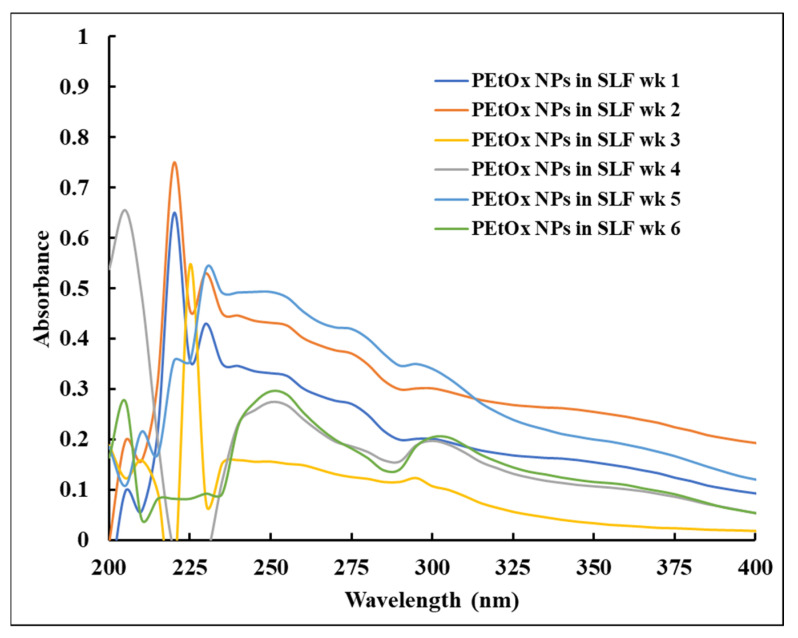
UV–Vis spectra of PEtOx NPs at different time intervals incubated in SLF. SLF was used as the reference.

PEtOx NPs incubated in SLF at 37 °C were also studied in the UV–Vis spectrophotometry, and the spectra are presented in Figure 15. The sharp characteristic peak of PEtOx NPs shifted from 215 nm to 220 nm after one week of incubation. Moreover, the distinct weak peak at 225 nm also moved to 230 nm, which indicates the NPs broke down or digested after one week (Figure 15). However, the typical peak of the free polymer was still present at 205 nm, indicating some NPs might return to their original polymeric form or the peak was from the free PEtOx polymer. After four weeks of incubation, the characteristic solid peak of PEtOx powder appeared at 205 nm, which confirmed that the NPs were digested and returned to their original structure. Similar findings were observed until the end of the incubation period. The findings revealed that the PEtOx NPs started to break down after one week of incubation, and complete digestion occurred after four weeks. The findings also match the result of FTIR and SEM analysis. However, in our previous study, UV–Vis spectra did not support the conclusions of FTIR and SEM when we studied the digestibility of the chitosan in the lysozyme solution [35]. Although all these three instruments have different principles and sensitivity, it is not easy to draw a final statement getting different results from the various analyses. However, the findings in this study are supported by all the analytical instruments.

The findings of this study indicate that the CIP-loaded PEtOx NPs are promising for lung delivery and could effectively control respiratory pathogens. Moreover, the developed formulation is negligible or minimal-toxic on the healthy and diseased bronchial epithelial cells; therefore, it is anticipated that it is safe to be used in pulmonary drug delivery. The prepared formulation has a good effect against respiratory pathogens at a very low dose and could reduce the suffering of LRTI patients. Therefore, the current high-dose antibiotic treatment of LRTIs could be replaced with a low-dose inhaled formulation. In addition, adding ZnO with the present formulation would improve the bactericidal effects and reduce the dose of antibiotics within the formulations. Therefore, the developed formulation is expected to be highly beneficial for patients suffering from LRTIs. The pulmonary delivery of low-dose formulations is expected to improve public health and positively impact the global economy.

### 2.10. Limitations of this Work

- The amount of free drug could not be detected in the supernatant of the centrifuged solution owing to the very low level of ZnO that was used to formulate the NPs. Therefore, the XPS analysis only detected the Zn associated with the NPs (surface and embedded). Further investigations using higher concentrations of ZnO are warranted.
- In this preliminary study, the pharmacokinetics study was not conducted to determine the fate of the undigested polymer in lung fluids. Further study is needed to have a complete understating of the fate of the undigested PEtOx polymer.

## 3. Materials and Methods

### 3.1. Materials

PEtOx with a molecular weight (MW) of 50 kDa and polydispersity index (PDI) 3–4; TA MW 1701 Da; high-performance liquid chromatography (HPLC) grade CIP powder MW 331.34 g/mol (assay: ≥98.0%); ZnO NPs (particle size ≤ 40 nanometre (nm), dispersion in 20 wt. % in H_2_O; and lysozyme from chicken egg white (protein ≥ 90%) were purchased from Sigma-Aldrich Pty Ltd. (Castle Hill, NSW, Australia). All other solvents/reagents used were analytical grade.

### 3.2. Preparation of Blank and CIP-Loaded PEtOx NPs

The preparation of blank and CIP-loaded PEtOx NPs was carried out followed by our previously published article [42]. In brief, blank NPs were prepared by dissolving PEtOx (1% *w*/*v*) and TA (0.03%) separately in deionized water and then adding the TA solution dropwise to the PEtOx solution over a magnetic stirrer at 1000 rpm. Moreover, the CIP-loaded PEtOx NPs were prepared by adding CIP powder (0.125% *w*/*v*) into the TA solution and then dropwise addition to the PEtOx solution. Finally, the blank and CIP-loaded PEtOx NPs were centrifuged and freeze-dried (Alpha 1–4 LD plus Freeze Dryer) to obtain powder NPs.

### 3.3. Preparation of CIP-ZnO-Loaded PEtOx NPs

CIP-ZnO-loaded PEtOx NPs were prepared by modifying the method described above. At first, 0.4 millimolar (mM) ZnO solution was prepared in deionized water based on the literature value of zinc homeostasis against the clinical strain of *P. aeruginosa* [69]. ZnO-PEtOx NPs were prepared by dissolving PEtOx (1% *w*/*v*) and TA (0.06% *w*/*v*) separately in ZnO solution by ultrasonication for 10 min. Then, the TA solution was added dropwise to the PEtOx solution at a stirring speed of 1000 rpm to form the NPs in a co-assembly reaction. CIP-ZnO-PEtOx NPs were prepared by dispersing CIP powder in the TA solution (0.125% *w*/*v*) and then adding dropwise to the PEtOx solution stirred at 1000 rpm. Finally, the NPs were separated by centrifugation at 6000 rpm and freeze-dried to obtain powder NPs.

### 3.4. Detection of Zn by XPS

XPS (Perkin-Elmer Corporation, MN, USA) was performed using a Kratos Axis Supra with aluminium k-alpha radiation (1486.7 eV). The spectrometer was operated in FAT mode. Survey scans were acquired using an analyser pass energy of 160 eV to ensure maximum sensitivity. High-resolution scans were performed with a pass energy of 20 eV to improve energy resolution at the expense of sensitivity. The instrument’s work function was calibrated to place the 4f 7/2 line of gold at a binding energy of 84.0 eV, and the instrument range was tuned to ensure the binding energy of the 2p 3/2 line of copper at 932.6. Charge compensation was performed using the in-built Kratos charge neutralizer, producing an offset of approximately −3 eV of binding energy, which was corrected by rigidly shifting all spectra to a known reference level.

### 3.5. Particle Size and Size Distribution of Blank and CIP-Loaded PEtOx NPs

The average particle size of blank and CIP-loaded PEtOx NPs was confirmed by dynamic light scattering (DLS) measurement using a Zetasizer Nano ZS 90 (Malvern Instruments, Worcestershire, UK). The NPs were dispersed in deionized water by ultrasonication for 5 min before the measurement. The measurement was carried out at the scattering angle of 90° and refractive index (RI) of 1.52 for PEtOx. All samples were analysed in triplicate at 25 °C.

### 3.6. Bacterial Culture

The bactericidal activity of the formulated NPs was studied against clinical strains of Gram-positive methicillin-resistant *S. aureus* (MRSA, USA 300) and Gram-negative multi-drug-resistant *P. aeruginosa* Trent [70]. These two respiratory bacteria were obtained from the Institute of Infection and Global Health, University of Liverpool, UK. Gram-negative and Gram-positive bacterial strains were selected to evaluate the permeability of the formulated NPs into the bacterial outer membrane. The bacterial strains were newly subcultured onto a horse blood agar (HBA) medium from the stock culture preserved at −80 °C. Then, the cultured plates were incubated at 37 °C for 24 h. A single bacterial colony was isolated from the subcultured bacteria colonies and suspended in sterile PBS (pH 7.3 ± 0.1) at compatible turbidity with 0.5 MacFarland.

### 3.7. Bactericidal Assay

The bactericidal assay was carried out using the previously described agar dilution method with some modifications [71,72]. The assay was also accountable to the recommendations of the National Committee for Clinical Laboratory Standards (NCCLS) document M07-A10 [73]. In brief, the CIP-loaded PEtOx and CIP-ZnO-loaded PEtOx NPs with various concentrations of CIP (4, 8, 16, and 32 µg/mL, *n* = 2 for each concentration) were applied to determine the bactericidal efficacy of the formulated NPs. The NPs were suspended in PBS and kept over a magnetic stirrer for seven days at 100 rpm with a temperature of 37 °C to allow adequate time to release the maximum amount of CIP from the polymeric matrix. The applied amount of NPs for drug release was determined based on our previous findings of drug loading and drug release studies, and the concentrations of the released CIP were determined by the HPLC method [42]. After seven days, the released CIP in PBS solutions were separated by centrifugation, sterilized by filter sterilizer, and mixed with MHA medium in a 1:9 ratio. Then, the mixture was spread onto a sterile bacterial culture plate. After MHA solidification, two holes of 5 mm diameter were punched by a sterile cork-borer. Then, 20 µL bacterial suspension of 0.5 MacFarland concentration was applied to each hole. Finally, the culture plates were incubated for 18 h at 37 °C. Pure CIP was used as the positive control with the concentration based on the literature values of minimum inhibitory concentration (MIC) of CIP against *S. aureus* (32 µg/mL) [74] and *P. aeruginosa* (32 µg/mL) [75]. MHA medium was used as the negative control. Raw PEtOx, blank PEtOx NPs, ZnO NPs, and ZnO-loaded PEtOx NPs were also investigated in this study to evaluate the bactericidal effects of the polymer, its NPs without drug, ZnO NPs, and ZnO-PEtOx conjugates.

The bacterial growth on the plates was visible after the incubation period. Then, the bacterial growth zone was measured edge to edge in diameter on the culture plates.

### 3.8. Cell Culture

Two different types of cells were used to study the cytotoxicity and inflammatory effect in response to blank PEtOx, CIP-loaded PEtOx NPs, and CIP-ZnO-loaded PEtOx NPs: airway epithelial cells as representative of structural cells and macrophages as representative of immune cells, also known as the first line of immune defence. Cryopreserved adult airway epithelial cells from a healthy donor (NHBE; Lonza), a donor with COPD (DHBE; Lonza), and an airway epithelial cell line derived from patients with CF (CFBE41o-) were cultured in an air–liquid interface (ALI) on 24-well plates in a humidified incubator in an atmosphere of 5% CO_2_/95% air at 37 °C. PneumaCult™-Ex Plus medium was used as a cell culture medium for NHBE and DHBEs; minimum essential medium (MEM) with 10% heat-inactivated fetal bovine serum (FBS) was used to culture CFBE41o- cell line. Monocytes, the precursor cells of macrophages, were sorted from buffy coats of HCs obtained from the Australian Red Cross Blood Service using CD14+ magnetic beads (Miltenyi). These CD14+ monocytes were differentiated into macrophages by 6-day stimulation with rhGM-CSF (50 ng/mL) in RPMI-1640 supplemented with 10% heat-inactivated FBS, 1% penicillin–streptomycin–fungizone (Lonza). Respiratory linings are enriched with GM-CSF; therefore, GM-CSF differentiated macrophages represent respiratory macrophages [76]. Healthy macrophages treated with CFTR inhibitor, CFTR_Inh_-172 (C-172), mimic various CF macrophage phenotypes, thereby named CF-like macrophages [77]. Structural and immune cells from healthy and diseased conditions were used to investigate the effects of the formulated compounds on chronic lung disease conditions.

### 3.9. Evaluation of Cellular Viability by LDH Assay

LDH release was quantified to determine cell death in response to the formulated compounds. A previously described method with some modifications was used to evaluate the cell viability [78]. All the cell types were seeded at 10,000 cells/well in 24-well plates and treated when confluent. The blank PEtOx and CIP-loaded PEtOx were dispersed in a 500 µL cell culture medium at 32, 128, or 2048 µg/mL. ZnO, ZnO-loaded PEtOx, and CIP-ZnO-loaded PEtOx NPs at the same concentrations were dispersed in PneumaCult™-Ex Plus medium and tested only using NHBE cells. After 24 h of exposure, 50 µL medium was removed from each well and mixed with 50 µL LDH storage buffer according to the manufacturer’s protocol (Promega LDH-Glo^TM^ Cytotoxicity Assay J2380 and J2381). LDH detection was performed according to the manufacturer’s instructions, and absorbance at 490nM was quantified using a CLARIOstar Plus (BMG Labtech, Ortenberg, Germany) microplate reader. Relative cell viability was determined from the equation: viability (%) = (absorbance of treated cells–absorbance of blank medium) / (absorbance of untreated cells–absorbance of blank medium) × 100. The blank medium was used as the control of a vehicle-only cell (negative), and 10% Triton X-100 was used as the positive control.

### 3.10. IL-8 Release

IL-8, as an indicator of induced inflammation, was quantified in response to CIP-loaded and non-CIP-loaded PEtOx NPs. Cells were exposed to the same concentrations of compounds as described above. After 24 h, 100 µL supernatant from each well was used to quantify IL-8 using alphaLISA (Perkin Elmer, Boston, MA, USA) as per the manufacturer’s instructions.

### 3.11. In Vitro Digestibility Study

PEtOx powder and PEtOx NPs were studied in an in vitro digestibility study using SLF. The SLF was prepared according to our previous study [35]. In brief, PBS was prepared in Milli-Q water, and lysozyme crystal (0.2 mg/mL) was dissolved in the prepared PBS solution (pH 7.4) to mimic the maximum lysozyme concentration in lung fluid. PEtOx powder and PEtOx NPs have suspended separately in the SLF (1.0 mg/mL) solution by sonicating for 5 min. All samples were incubated at 37 °C on an orbital shaker at 100 rpm. The suspensions were tested to determine the pH weekly, and aliquots were taken and replaced with freshly prepared SLF. The suspensions were centrifuged at 6000 rpm for 30 min to separate the supernatant, and the remaining was freeze-dried to obtain a dry powder.

### 3.12. ATR-FTIR

ATR-FTIR spectra were obtained from all SLF-treated freeze-dried powder samples using a Thermo iS50 FTIR spectrometer (Nicolet, Madison, WI, USA). The instrument was equipped with a single reflection diamond crystal with an angle of incidence of 40° and a deuterated triglycine sulphate (DTGS) detector. Spectra were collected using 8 cm^−1^ resolution, 64 scans, and a range of data collection 4000–400 cm^−1^. OMNIC analytical software was used to analyse the data collected from the spectra (Nicolet Instrument Corp., Version 9.2, Madison, WI, USA).

### 3.13. Morphology Analysis by SEM

The surface morphology of SLF-treated freeze-dried powder samples was investigated by SEM (Tescan Mira3, Brno, Czech Republic). Samples were prepared by scattering powder on carbon adhesive tape mounted to the surface of an aluminium stub. Then, a blowing nitrogen gun was used to remove the access particles from the carbon adhesive tape. Finally, the samples were coated with a 5 nm thick layer of gold. The samples were observed in SEM at a 5 kV stepping-up voltage with a working distance of 8.08 mm.

### 3.14. UV–Vis Spectroscopy

Using a UV–Vis spectrometer (Cary 60 UV–Vis Spectrophotometer, Santa Clara, CA, USA), the solutions of original PEtOx powder and its NPs were analysed in the absorbance range of 200–400 nm to understand the digestibility of the polymer and its NPs in PBS and artificial lung fluid solutions. Initially, PEtOx powder was dissolved in PBS (pH 7.4) solution (16 µg/mL) to obtain the UV–Vis characteristic peaks of the original polymer. Similarly, PEtOx NPs were dissolved in PBS solution to determine the distinct peaks of the NPs. The original lysozyme crystals dissolved in PBS (pH 7.4) solution to obtain a concentration of 0.2 mg/mL the UV–Vis spectroscopy analysis was carried out to determine whether the lysozyme solution interferes with the characteristic peaks of the polymer or their NPs. Blank PBS was used as a reference in these experiments. Finally, liquid samples from the digestibility study were analysed using the same UV–Vis spectrophotometer and the blank SLF solution was used as the reference.

### 3.15. Statistical Analysis

All the experimentations were carried out in triplicate, and the results are expressed in mean ± standard deviation (SD). One-way analysis of variance (ANOVA) was applied as the statistical analysis between the two groups. Probability (*p*) values of < 0.05 were considered significant differences.

## 4. Conclusions

This study demonstrated that CIP-loaded PEtOx NPs retained their bactericidal effects after encapsulation and drug release against the clinical strains of *S. aureus* and *P. aeruginosa*. CIP-loaded PEtOx NPs revealed significantly improved (*p <* 0.05) bactericidal effects in combination with the traces of ZnO against both clinical strains. CIP-loaded PEtOx NPs appeared to be biocompatible on NHBE cells. COPD cells and CF cell lines showed a low cytotoxicity level against CIP-loaded PEtOx NPs. However, IC_50_ values of COPD cells (0.103 mg/mL) and CF cell lines (0.514 mg/mL) suggest that MIC (32 µg/mL) of the respiratory pathogens could be achieved safely by this formulation. Nevertheless, healthy and CF-like macrophages demonstrated a high level of cytotoxicity against CIP-loaded PEtOx NPs. Therefore, further studies are warranted to confirm the absolute safety of this formulation. ZnO and ZnO-PEtOx NPs demonstrated excellent biocompatibility on NHBE cells. This study also demonstrated the in vitro digestibility of PEtOx powder and PEtOx NPs in SLF (pH 7.4). ATR-FTIR, SEM, and UV–Vis analysis showed that PEtOx NPs are digestible in SLF (0.2 mg/mL lysozyme solution). Moreover, the findings revealed that the PEtOx polymer is not digestible in SLF even after six weeks of incubation. However, PEtOx could be used in pulmonary drug delivery because of its minimal-toxic effects on the bronchial epithelial cell and cell lines. The outcomes of this study also revealed that traces of ZnO in combination with CIP-loaded PEtOx NPs would be a promising bactericidal agent against biofilm-forming respiratory pathogens resistant to most antibiotics. Therefore, it can be concluded that PEtOx polymer could be considered an efficient drug delivery carrier in respiratory linings, and CIP-loaded PEtOx NPs with traces of ZnO could be a promising addition to the management of LRTIs with reduced systemic toxicity; however, further studies of in vivo trials are warranted to draw a complete conclusion.

## Data Availability

Data are contained within the article.

## Supplementary Materials

The supporting information can be downloaded at: https://www.mdpi.com/article/10.3390/ijms24054532/s1.
