# Supplementary figures and images for: Cytotoxic and Bactericidal Effects of Inhalable Ciprofloxacin-Loaded Poly(2-ethyl-2-oxazoline) Nanoparticles with Traces of Zinc Oxide
Source: Int J Mol Sci. 2023 Feb 25;24(5):4532. doi: 10.3390/ijms24054532 (PMC10002581; doi:10.3390/ijms24054532)

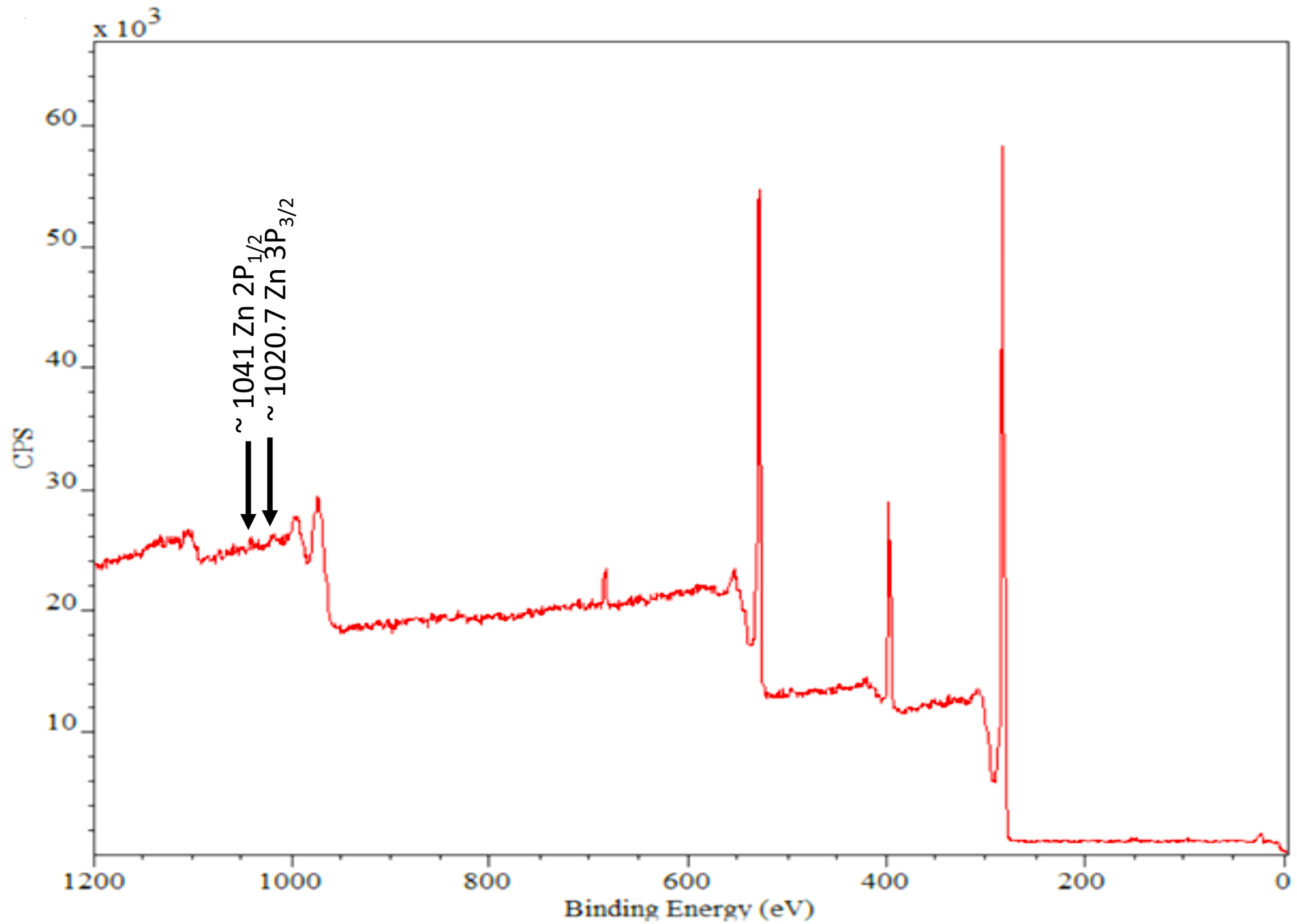

Supplement: Supplementary file 1 [file ijms-24-04532-s001.zip › ijms-2220134-supplementary Figure S1.pdf]
